# Genomic Instability in Newborn with Short Telomeres

**DOI:** 10.1371/journal.pone.0091753

**Published:** 2014-03-12

**Authors:** Jennifer Moreno-Palomo, Amadeu Creus, Ricard Marcos, Alba Hernández

**Affiliations:** 1 Grup de Mutagènesi, Departament de Genètica i de Microbiologia, Facultat de Biociències, Universitat Autònoma de Barcelona, Campus de Bellaterra, Cerdanyola del Vallès, Spain; 2 CIBER Epidemiología y Salud Pública, Instituto de Salud Carlos III, Madrid, Spain; Université de Sherbrooke, Medicine, Canada

## Abstract

Telomere length is considered to be a risk factor in adults due to its proved association with cancer incidence and mortality. Since newborn present a wide interindividual variation in mean telomere length, it is relevant to demonstrate if these differences in length can act also as an early risk indicator. To answer this question, we have measured the mean telomere length of 74 samples of cord blood from newborns and studied its association with the basal genetic damage, measured as the frequency of binucleated cells carrying micronuclei. In addition, we have challenged the cells of a subgroup of individuals (N = 35) against mitomycin-C (MMC) to establish their sensitivity to induced genomic instability. Results indicate that newborn with shorter telomeres present significantly higher levels of genetic damage when compared to those with longer telomeres. In addition, the cellular response to MMC was also significantly higher among those samples from subjects with shorter telomeres. Independently of the causal mechanisms involved, our results show for the first time that telomere length at delivery influence both the basal and induced genetic damage of the individual.

**Impact:**

Individuals born with shorter telomeres may be at increased risk, especially for those biological processes triggered by genomic instability as is the case of cancer and other age-related diseases.

## Introduction

Genomic instability syndromes in humans are usually characterized by high sensitivity to genotoxic compounds and a high incidence of cancer [1]. Such syndromes have been very useful to discover the many genes involved in recognizing, signaling and/or repairing DNA damage, DNA processing, cell cycle regulation, apoptosis and/or telomeric maintenance [2]. In addition to the dramatic effects that appear when these genes do not work properly, slight changes in their function due to genetic polymorphisms are also considered as individual susceptibility factors [3]–[5].

Telomere dysfunction has also been pointed out as leading to chromosome instability [6], [7]. This fact is based on the finding that cancer predisposition syndromes lead to telomere dysfunction and chromosome instability [8]. Increasing evidence also supports the importance of telomeres in DNA repair; thus, numerous reports indicate a strong relationship between telomeric proteins and those proteins involved in cellular responses to DNA damage. Defects in telomeric proteins lead to telomere dysfunction, genomic instability and cancer predisposition [9]–[11].

During somatic-cell replication, telomere length progressively shortens because of the inability of DNA polymerase to fully replicate the 3′ end of the DNA strand [12]. Once a critically short telomere length is reached, the cell is triggered to enter replicative senescence, which subsequently leads to cell death [13]. Conversely, in germ cells and other stem cells that require renewal telomere length is maintained by the enzyme telomerase. Both longer and shorter telomere lengths are associated with increased risk of certain cancers [14], and reactivation of telomerase is a common requirement for oncogenic progression. Therefore, telomere length is an important determinant of telomere function.

At the individual level, telomere length decreases with age and somatic cell telomere length values highly correlate within the same individual, although with some extent of variation [15], [16]. At birth, human telomeres are typically 8–14 kb long, but with a substantial interindividual heterogeneity [17], [18]. Although differences in maternal-placental immune and endocrine processes during gestation have been proposed to affect the telomere biology [19], it is generally accepted that the initial setting of telomere length could affect the subsequent attrition rate and, possibly, their consequences [20].

Short telomeres have been suggested to be a potential cancer predisposition factor as it is indicative of increased genomic instability. Indeed, telomere lengths have been proposed to be used for identifying high-risk population groups [21]. In this context, the frequency of micronuclei (MN) in lymphocytes is accepted as a sensitive indicator of genetic damage and is considered a good surrogate biomarker of cancer risk [22]. MN are defined as small chromatin bodies appearing in the cytoplasm due to the condensation of acentric chromosome fragments or by whole chromosomes, lagging behind during cell division. Thus, it is one of the few biomarkers that allow for the simultaneous evaluation of both clastogenic and aneugenic effects on the whole genome. In addition, the use of the MN assay to evaluate the individual sensitivity to genotoxic agents has been reported to be a useful tool for determining the intrinsic genomic instability of different population groups, such as that of cancer-prone individuals [23].

Considering the existent telomere length interindividual variability among newborns, the present work aims to determine whether there is an association between telomeric length and the spontaneous level of genetic damage in newborn. In addition, the exposure of cord blood lymphocytes to the well known genotoxic compound mitomycin C will allow us to determine whether this telomeric length is associated with an increased level of genomic instability.

## Materials and Methods

### Cord blood samples

Fresh cord blood samples from 74 newborn were obtained from the Cord Blood Bank in Barcelona. Lymphocytes were isolated from cord blood samples by the Lymphoprep sedimentation method. The study was approved by the Ethics Committee of our university (Universitat Autónoma de Barcelona). All samples were collected and further manipulated in accordance with ethical standards.

### Telomere length measurement

DNA was extracted from freshly isolated lymphocytes of cord blood samples using a standard phenol-chloroform method and dissolved in TE buffer for Terminal Restriction Fragment (TRF) measurement. Prior to the analysis, all DNAs were quantified with the Nanodrop spectrophotometer and only those with a 260/280 ratio in the range of 1.8–2.2 were included in the study Also, all samples were resolved in a 0.6% agarose gel electrophoresis at 100 V for 45 min in order to ensure DNA integrity.

TRF size was determined by a conventional enzymatic digestion and Southern hybridization method using a telomeric probe from the TeloTAGGG Telomere Length Assay (Roche, Manheim, Germany). Briefly, a total of 2 μg of DNA were digested with a mixture of the restriction enzymes *Hinf*I and *RsaI* according to the manufacturer instructions and then resolved in a 0.7% agarose gel electrophoresis. After electrophoresis, DNA was transferred from the agarose gel onto a nitrocellulose membrane and hybridized with the telomeric probe (TTAGGG)_4_ labeled with digoxigenin (DIG). The probe was detected with an anti-DIG-AP antibody. A chemiluminescent signal was recorded on film within the linear range and further analyzed in an image densitometer. The average TRF length was calculated as the weighted mean of the optical density as described by Petersen et al. [24]. TRF length measured by this method is composed of distal telomeric (TTAGGG) repeats and subtelomeric sequences as both are recognized by the telomeric probe used.

### Lymphocyte cultures and MN analysis

Lymphocyte cultures from newborn cord blood samples were established by adding 0.5×10^6^ isolated lymphocytes to 4.5 mL of RPMI 1640 medium supplemented with 15% heat-inactivated fetal calf serum, 1% antibiotics (penicillin and streptomycin) and 1% L-glutamine (all provided by Gibco Life Technologies, Paisley, UK). Lymphocytes were stimulated by 1% phytohaemagglutinin (Gibco) and incubated for 72 h at 37 °C.

A cytochalasin B (Cyt-B, Sigma, St. Louis, MO, USA) solution prepared in dimethylsulphoxide at a concentration of 6 μg/mL was added to the cultures 44 h later to arrest cytokinesis. After 72 h of incubation, the cultures were harvested by centrifugation at 800 rpm for 8 min. Next, cultures were washed once in RPMI 1640 medium to preserve cell cytoplasm, and a mild hypotonic treatment (2–3 min in 0.075 M KCl at 4 °C) was carried out thereafter. Cells were centrifuged and a methanol-acetic acid (3∶1 vol/vol) solution was gently added. This fixation step was repeated twice, and the resulting cells were resuspended in a small volume of fixative solution and dropped onto clean slides. Finally, slides were stained with 10% Giemsa (Merck, Darmstadt, Germany) in phosphate buffer (pH 6.8) for 10 min. To determine the frequency of binucleated cells with micronuclei (BNMN) and the total number of MN, a total of 1000 binucleated cells with well preserved cytoplasm (500 per replicate) were scored for each subject. As for MN formation it is required that cells undergo replication, the cytokinesis-block proliferation index (CBPI) was calculated in order to ensure the proliferative state of the cultures [25]. In this case, 500 lymphocytes were scored to evaluate the percentage of cells with one to four nuclei.

In the subset of 35 samples challenged with mitomycin-C, 0.2 μM of MMC was added to the cultures for 48 h. All the following procedures were as indicated above.

### Statistical methods

Student-*t* test was performed for comparisons between groups on data with normal distribution (KS test). Pearson's test was used to evaluate correlations between the different variables, and linear regressions were performed to establish the association between telomere length and basal or induced BNMN.

All reported probability values were two-tailed and the criterion for significance was set at *P*<0.05. The statistical software used for the data analyses was STATISTICA (StatSoft, Inc. 1996, Tulsa, OK, USA) on a Pentium PC-compatible.

## Results

**Figure 1:**
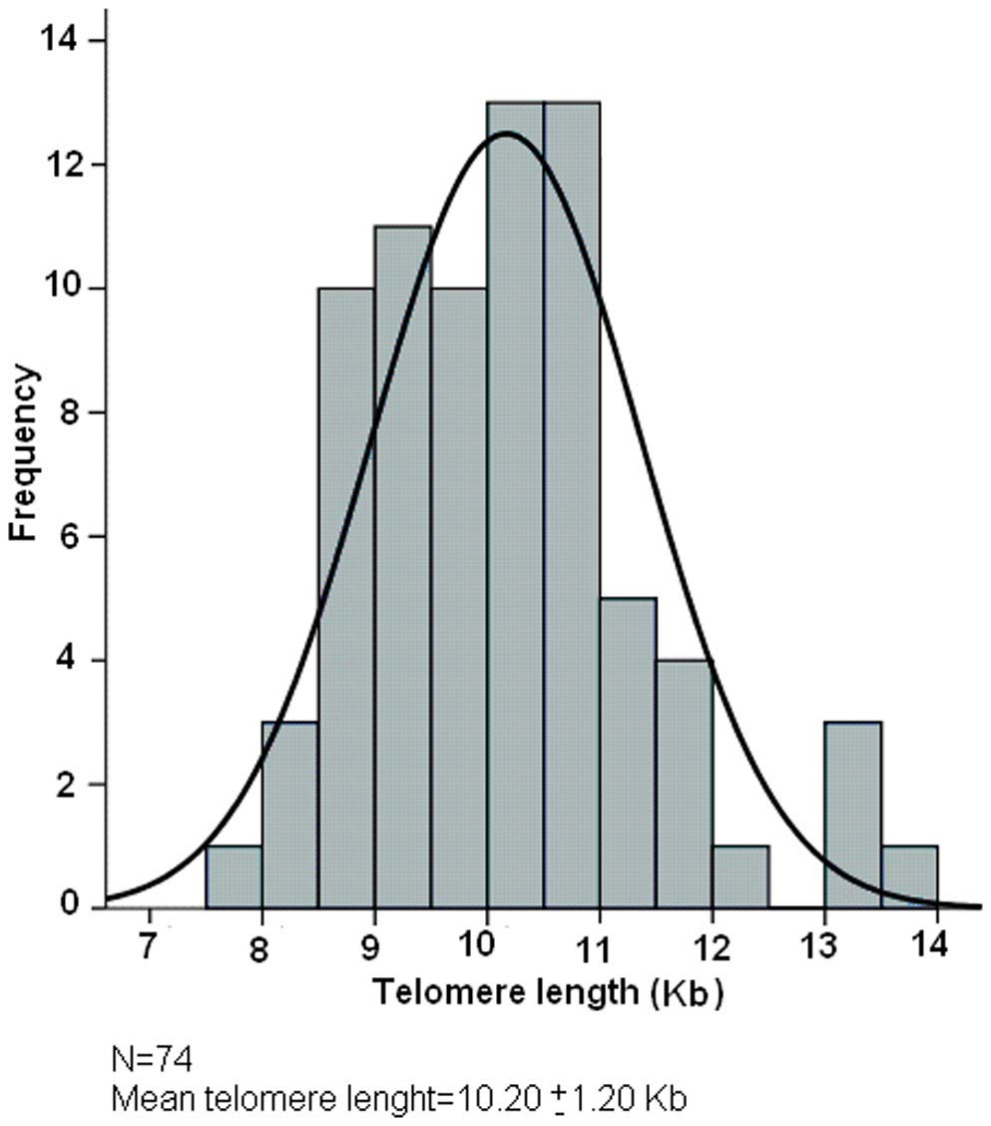
Telomere length distribution in the newborn population (N = 74). As observed, TRF length is heterogeneous from birth.

**Figure 2:**
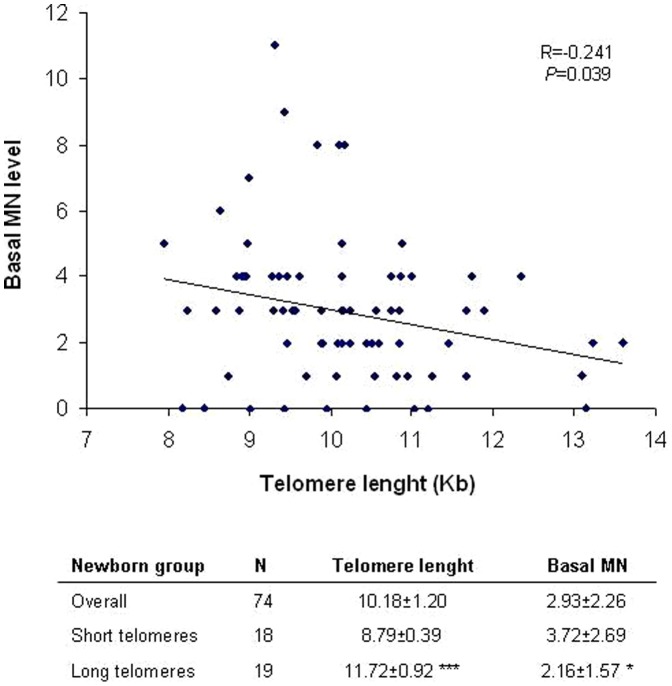
Correlation between TRF length and newborn baseline genetic damage. A negative significant association is observed, were individuals with shorter telomeres present significantly higher levels of DNA damage. The mean ± SEM values of telomere length and basal DNA damage are also indicated for the overall population (N = 74) and for the individuals with shorter (N = 18) and longer (N = 19) telomeres. Individuals with shorter telomeres show significant higher level of DNA damage. Student *t*-test for long *vs* short telomere groups; *P<0.05, **P< 0.01.

The measurement of the TRF lengths of the 74 sampled newborn showed a wide interindividual variability with a TRF average length of 10.20±1.20 Kb, ranging from 8 to 13.6 Kb. The distribution of these data is indicated in Figure 1, showing a behaviour not departing from normality (*P* = 0.20). When the overall population was classified in quartiles according to TRF length, important differences in TRF length were observed between the most extreme groups (see Figure 2). Thus, the group with longer telomeres (N = 19, 11.72 ± 0.92 Kb) was significantly different (*P*≤0.001) from the group with shorter telomeres (N = 18, 8.79 ± 0.39 Kb), confirming that telomere lengths of newborn are heterogeneous from birth.

When the level of basal genetic damage was calculated -as measured by the frequency of binucleated cells with micronuclei (BNMN)- the mean value obtained was 2.93 ± 2.26, with values ranging from 0 to 11 BNMN (figure 2). Figure 2 also shows the individual values of basal BNMN plotted against the individual TRF values. A negative correlation is observed (R = −0.241), where those individuals with shorter telomeres present significantly higher values of BNMN (*P* = 0.039). The same is observed when comparing the mean MN values of the group of individuals with shorter and longer telomeres; the basal MN level of individuals with shorter telomeres is 3.72 ± 2.69 while individuals with longer telomeres present a mean MN value of 2.16 ± 1.57 (*P* = 0.037). CBPI values were homogeneous along the different groups, indicating that the proliferative state of cord blood samples used for the MN assay was similar (data not shown).

**Figure 3:**
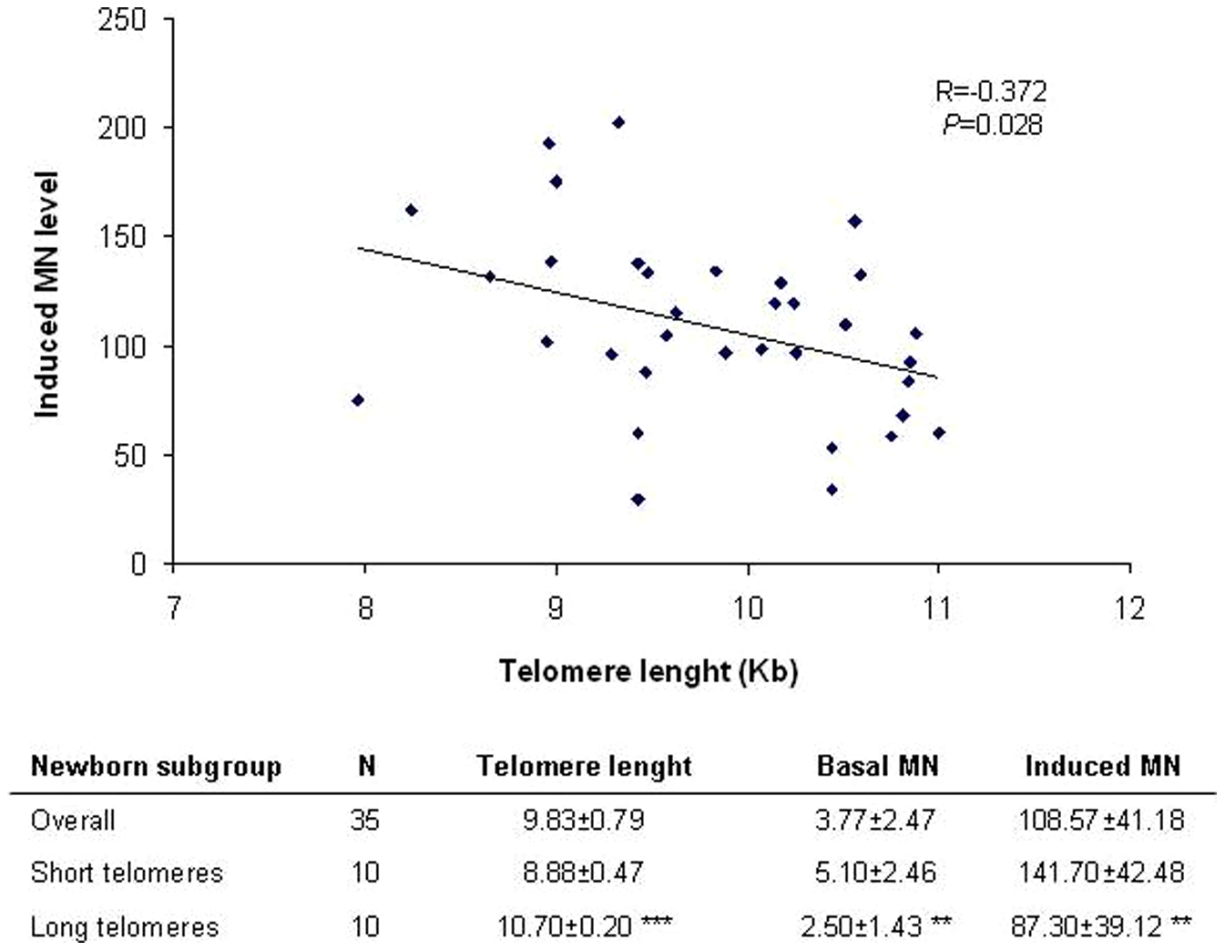
Correlation between TRF length and newborn induced genetic damage. A subgroup of 35 cord blood samples were selected and challenged to MMC treatment to establish their sensitivity to induced genomic instability. As observed, individuals with shorter telomeres present higher levels of MMC-induced genetic damage. The mean ± SEM values of telomere length, basal and induced DNA damage are also indicated for the overall population (N = 35) and for the individuals with shorter (N = 10) and longer (N = 10) telomeres. Individuals with shorter telomeres show significant higher level of basal and induced DNA damage. Student *t*-test for long vs. short telomere groups; **P<0.01, ***P<0.001.

A subgroup of 35 individuals from the population was selected in order to determine newborn individual genomic instability. This was achieved by performing a challenging experiment with MMC, where cord blood lymphocytes were exposed *in vitro* to the established genotoxic compound. The TRF length characteristics of the selected subgroup of individuals are similar to that of the former population, where wide TRF interindividual variability was observed. Significant differences were found when the individuals of the subgroup with more extreme TRF length values were compared (Figure 3). After challenging the cells with MMC, larger increases in the frequency of induced BNMN were found among those individuals with shorter telomeres (141.70 ± 42.48 *vs* 87.30 ± 39.12, respectively; *P* = 0.01; Figure 3). Moreover, a negative correlation was observed (R = 0.372) between TRF values of the subgroup and the MMC-induced genomic instability (*P* = 0.028; Figure 3). Basal MN level of individuals with longer telomeres were also found to be lower than that of individuals with shorter telomeres (2.50 ± 1.43 *vs* 5.10 ± 2.46, respectively; *P* = 0.01; Figure 3) as was previously found in the former population.

As a whole, our results indicate an interesting association between newborn TRF length and BNMN values, both at baseline and induced level.

## Discussion

Important associations between telomere length, chromosome instability and cancer have been extensively reported [26]. Nevertheless, most of those results came from case-control studies that make it difficult to establish definitive causal relationships. To solve this problem, different prospective studies have been carried out showing statistically significant inverse relationships between telomere length and both cancer incidence and mortality, as the Bruneck Study in Italy [14] and others summarized in the recent review from Prescot et al. [26]. Nevertheless, those cancers more influenced by smoking and inflammatory processes show closer associations than, e.g., breast cancer, indicating a complex background. Since chromosome aberrations have been associated with tumor aggressiveness [27], short telomeres may predict tumors displaying higher degrees of genomic instability. In fact, we have previously demonstrated that individuals with short telomeres are more sensitive to ionizing radiation [28]. In that study, carried out with healthy individuals aged around 21 years, we observed the higher induction of chromosome breaks in those individuals with shorter telomeres, when they were challenged with 3.5 Gy of ionizing radiation. All these studies show that in healthy subjects the individual levels of telomere length, regardless of their origin, act as a real risk factor for developing pathologies linked to genomic instability.

Interestingly, in our study we show that this risk factor is something not only acquired during adulthood, but also during prenatal development. Thus, depending on the processes occurring during the in uterus developmental stage, the newborn will present a characteristic telomere length at delivery that could affect the risk of suffering from those pathologies related to genome instability, encouraging TRF length as an early-risk indicator to be used in newborn. An important question which still remains unknown is the source of the existing telomere length heterogeneity, and also its associated consequences, where genetic and environmental factors are presumably involved. Indeed, in spite of the high heritability of telomere length [29], the known genetic variants determined until now [5] explain only a limited proportion of the observed variability [7], [30]. The wide variability of TRF length observed in our newborn study agrees with previous publications [16] and is consistent with the view that variations observed in adults are largely due to genetic and environmental determinants that start exerting their effects in uterus [17]. This puts emphasis on the importance of the intrauterine environment acting during pregnancy as a modulator of the telomere length at birth. In this direction, a wide variability in the frequency of chromosome aberrations has been observed in newborn associated to the levels of polycyclic aromatic hydrocarbons (PAHs) measured during pregnancy [30]. PAHs were measured prenatally during the third trimester of pregnancy by personal air monitors carried out by the mothers during a 48 h period, indicating that such exposure can lead to sub-clinical genetic effects at birth. In addition to the exposure to environmental contaminants, diet is also considered as a factor. In fact, a cross-sectional analysis has demonstrated a positive association between the use of dietary multivitamin antioxidants and telomere length [31], as well as with the levels of dietary fiber intake [32]. Nevertheless, a recent study showing that high intake of vegetables and beta-carotene is positively associated with telomere length failed to show chromosome instability [33]. Unfortunately, no nutritional studies involving telomere length in humans have been performed, but Tarry-Adkins et al. [34] demonstrated a telomere shortening effect via alterations in the antioxidant defense capacity using a rat model subjected to gestational protein restriction.

Other factors such as psychosocial stress are currently being explored as new important factors modulating telomere length at birth. These factors constitute what is known as a fetal or developmental programming of health [35]. Maternal psychological stress during pregnancy has been found to have a significant effect on the newborn leukocyte telomere length [36]. but these type of studies need to be extended prior to consider any kind of biological plausibility between stress conditions and effects on telomere biology. Also, factors such as maternal and placental hormones and inflammatory and oxidative stress mediators entering the fetal circulation have been proposed as mechanisms altering fetal metabolism [17], [37], [38]. Such factors should be included as part of the biological stress measurements in cohort studies to better understands the underlying mechanisms of telomere attrition during gestation. Despite of the questions that remain to be addressed, overall results are interesting enough to accept that telomere biology is a mechanism that responds to stress mechanisms, at least during critical periods of fetal development.

In spite of the underlying mechanism affecting the final telomere length at birth, our results show for first time that the variability observed in a group of newborn affects not only their levels of basal genetic damage, but also how their lymphocytes are capable of responding to genotoxic insults during childhood and possibly during their adult life-span. According to our observations, telomere length at delivery can be considered a risk factor. An interesting question that remains to be solved is what percentage of genetic and/or environmental factors during pregnancy modulate the final average telomere length at delivery and, consequently, the genetic risk of the newborn.
